# Concerns about clinical efficacy and safety of warfarin in diabetic patients with atrial fibrillation

**DOI:** 10.1186/s12933-019-0818-0

**Published:** 2019-01-28

**Authors:** Sho-ichi Yamagishi

**Affiliations:** Department of Pathophysiology and Therapeutics of Diabetic Vascular Complications, Kurume University School of Medicine, Kurume, 830-0011 Japan

**Keywords:** Advanced glycation end products, Atrial fibrillation, Bone fracture, Diabetes, Vascular calcification, Warfarin

## Abstract

Atrial fibrillation (AF) is one of the most common arrhythmias in elderly people. The risk of thromboembolic stroke is increased in AF patients, especially those with diabetes. Anticoagulant therapy, such as warfarin and non-vitamin K oral anticoagulants (NOACs), is recommended for diabetic patients with AF. However, recent guidelines do not preferentially recommend NOACs over warfarin for diabetic patients. Variability of glycemic control in diabetic patients could affect the pharmacokinetics and anticoagulant activity of warfarin, therefore, the risk–benefit balance of warfarin is prone to be compromised in diabetic patients with AF. Furthermore, since warfarin inhibits the vitamin K-dependent gamma-glutamyl carboxylation of proteins, including osteocalcin and matrix Gla protein, use of warfarin may increase the risk of osteoporotic bone fracture and vascular calcification, both of which are the leading causes of morbidity that diminish the quality of life in diabetic patients. Even though the cost of NOACs is high, NOACs may be preferable to warfarin for the treatment of diabetic patients with AF.

## Background

A number of papers have suggested that diabetes is one of the risk factors for development of atrial fibrillation (AF) [1–3]. The Framingham Heart Study showed that comorbidity-adjusted risk for developing AF was 1.4 and 1.6 in diabetic men and women, respectively [1]. Meta-analysis of 7 prospective cohort and 4 case–control studies comprised of about 1,686,000 people revealed that hazard ratio for developing AF in type 2 diabetic patients was 1.39 compared with non-diabetic subjects [2]. Furthermore, a recent prospective study comprised of about 35,000 type 1 diabetic patients and age-, sex-, and birthplace-matched 175,000 controls also showed that patients with type 1 diabetes had a significantly higher risk for developing AF compared with controls; hazard ratios for AF in type 1 diabetic patients versus controls were 1.13 in men and 1.50 in women [3]. Since the excess risk for developing AF was larger in diabetic patients with poor glycemic control or a long disease duration, cumulative hyperglycemic exposure may partly contribute to new-onset AF in diabetic patients [1–3]. Oxidative stress and inflammation have also been shown to play a role in the pathogenesis of AF in diabetes [4, 5].

**Fig. 1:**
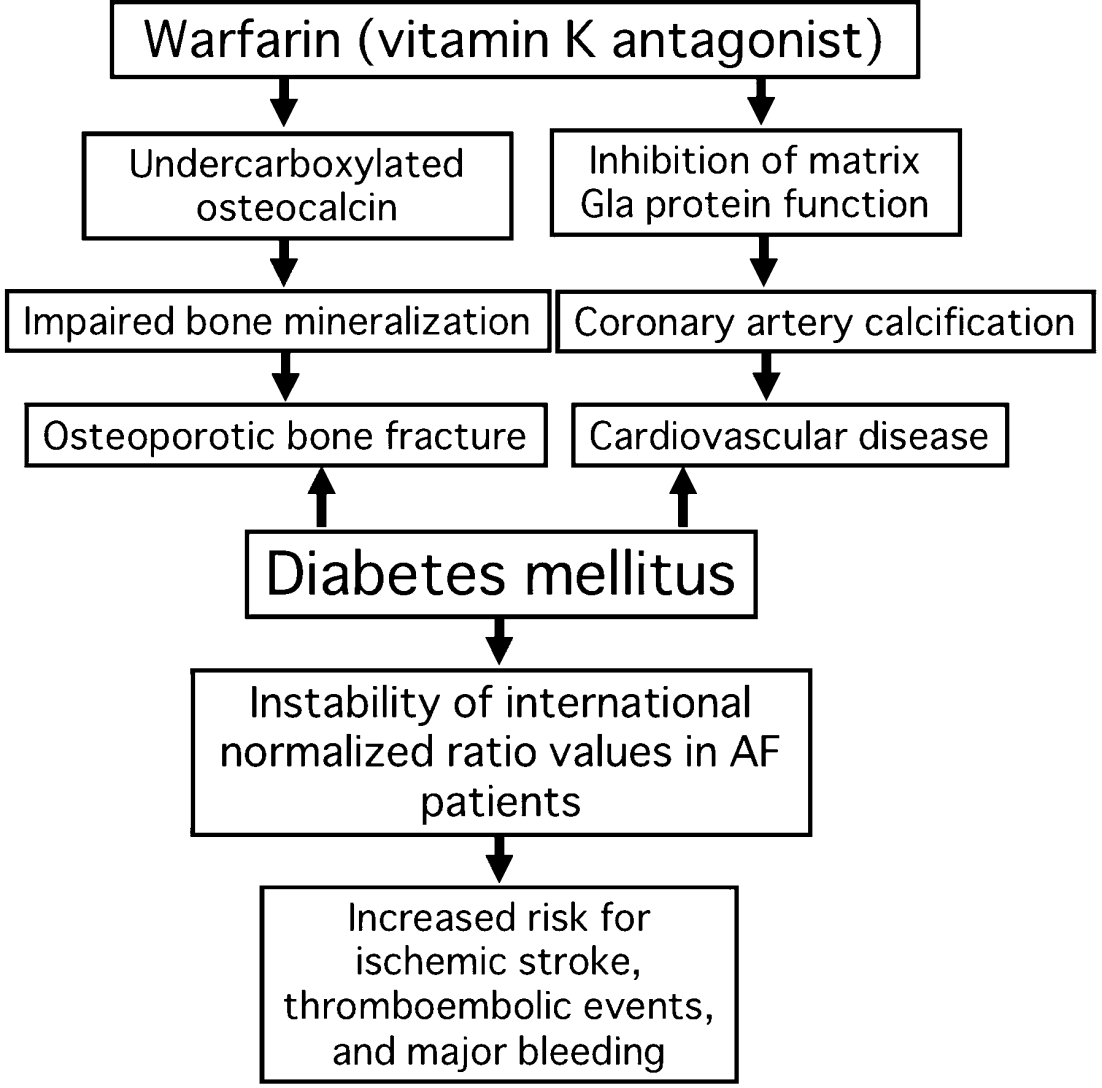
Clinical concerns about warfarin use in diabetic patients with AF. *AF* atrial fibrillation, *Gla* gamma-carboxyglutamic acid

CHA_2_DS_2_-VASc score is used for stroke risk stratification for AF patients in current guidelines, which is calculated by awarding 1 point each for congestive heart failure, hypertension, diabetes, presence of vascular disease, age 65–74 years, and female, and assigning 2 points for age ≥ 75 years and presence of prior stroke or transient ischemic attack [6]. Oral anticoagulant therapy, such as warfarin and non-vitamin K oral anticoagulants (NOACs) is recommend for AF patients with CHA_2_DS_2_-VASc score of 2 [6]. Among the CHA_2_DS_2_-VASc score components, diabetes is one of the stronger risk factors for ischemic stroke in AF patients; hazard ratios for ischemic stroke are 2.66 in both diabetic men and women compared with non-diabetic individuals [7]. Hyperglycemia is associated with enhanced thrombin formation in patients with diabetes or cardiovascular disease, which could increase the risk of thromboembolic events [8, 9]. Meta-analysis of four randomized clinical trials to compare the efficacy and safety of NOACs with warfarin revealed that NOACs significantly reduced stroke or systemic embolic events, intracranial hemorrhage, and all-cause mortality without the increased risk of bleeding except for gastrointestinal bleeding in a broad range of AF patients, including those with diabetes [10, 11]. However, recent guidelines do not preferentially recommend NOACs over warfarin for diabetic patients [6]. Here I would like to raise clinical concerns about the use of warfarin for diabetic patients with AF (Fig. 1).

Non-enzymatic glycation of amino groups of proteins has progressed under hyperglycemic conditions, which could alter the structure and function of various circulating and tissue matrix proteins, thus being involved in diabetes-associated complications, such as atherosclerotic cardiovascular disease and osteoporosis [12]. Compared with control albumin, glycated albumin or albumin purified from diabetic patients had the decreased binding affinity to warfarin with higher free fraction of this anticoagulant [13]. Therefore, variability of glycated albumin, a marker of short-term (2–3 weeks) glycemic control, may affect the pharmacokinetics of warfarin and its anticoagulant activity in diabetic patients. Indeed, when patients were stratified by quartile of international normalized ratio (INR) values out of range, presence of diabetes was independently associated with worst INR control in warfarin-treated AF patients [14]. The efficacy and safety of warfarin are totally dependent on the time in therapeutic range (TTR) of INR; 10% decrease in TTR is associated with about 10% rise in ischemic stroke and thromboembolic events [15]. Risk of major bleeding was significantly higher in AF patients receiving warfarin with TTR < 66% than those treated by NOACs [10]. These findings suggest that risk–benefit balance of warfarin is prone to be compromised in AF patients with diabetes.

Warfarin exerts anticoagulant effects by inhibiting the vitamin K-dependent gamma-glutamyl carboxylation of clotting factors II, VII, IX, and X [16, 17]. However, this type of posttranslational modification is also essential for the proper functioning of other gamma-carboxyglutamic acid (Gla) proteins, such as osteocalcin (bone Gla protein) and matrix Gla protein (MGP) [16, 17]. Osteocalcin-deficient mice have been shown to develop hyperostosis, whereas gamma-carboxylation confers a greater Ca-binding capacity to osteocalcin, playing a crucial role in normal bone mineralization [16, 17]. Therefore, warfarin may have deleterious effects on bone health. Circulating vitamin K levels were decreased in women with osteoporotic hip fracture and inversely correlated with incidence of vertebral fracture [16, 17]. High levels of undercarboxylated osteocalcin, a marker of low vitamin K status, were associated with the reduced lumber bone mineral density and predicted the increased risk of hip fracture in healthy women [16, 17]. Moreover, intake of vitamin K-rich food is restricted in AF patients receiving warfarin. In consistent with these findings, the risk of osteoporotic bone fracture was reported to be significantly increased in elderly patients with AF receiving long-term warfarin therapy compared with untreated subjects [18]. Meta-analysis revealed that the risk of bone fracture was significantly lower in AF patients receiving NOACs compared with warfarin [19]. As is the case with AF, osteoporotic bone fracture is increased in both type 1 and type 2 diabetic patients, especially in those with a long-term disease history [12]. Given the potential adverse effects of warfarin on osteoporotic bone fracture, NOACs may be safer than warfarin for the treatment of diabetic patients with AF.

MGP is a Ca-binding extracellular matrix protein with inhibitory effects on soft tissue calcification, which is mainly produced by chondrocytes and vascular smooth muscle cells [20–24]. While MGP-knockout mice displayed massive arterial calcification, warfarin, but not rivaroxaban induced vascular calcification in apolipoprotein E-deficient mice [21, 22]. Undercarboxylated and nonphosphorylated MGP levels predicted the risk of all-cause mortality and cardiovascular death in a general population [20]. Use of vitamin K antagonists (VKAs), such as warfarin, was associated with the increased risk for coronary artery calcification in patients irrespective of the presence or absence of AF; mean coronary calcification scores were significantly increased with the duration of VKA use [22, 23]. Moreover, supplementation of vitamin K was reported to significantly inhibit the progression of coronary artery calcification in elderly patients [25]. Since coronary artery calcification is more prevalent in diabetes and predicts future cardiovascular events and death [26], NOACs would seem preferable to warfarin for prevention of atherosclerotic cardiovascular disease. Further randomized controlled trials of NOACs vs. warfarin in diabetic patients with AF are warranted to address these clinical concerns about warfarin use.

## Conclusion

Given the instability of anticoagulant activity of warfarin and its potential deleterious effects on bone and vasculature, NOACs may be preferable to warfarin for the treatment of diabetic patients with AF.

## Abbreviations

AF: atrial fibrillation
NOACs: non-vitamin K oral anticoagulants
INR: international normalized ratio
TTR: time in therapeutic range
Gla: gamma-carboxyglutamic acid
MGP: matrix Gla protein
VKAs: vitamin K antagonists
